# Predicting Pathways into Criminal Behavior: The Intersection of Race, Gender, Poverty, Psychological Factors

**Published:** 2017

**Authors:** Lisa Christine Walt, Leonard A. Jason

**Affiliations:** Center for Community Research, DePaul University, Chicago IL

**Keywords:** Women, Substance Abuse Recovery, Criminal Justice Involvement, Arrest, Incarceration, Conservation of Resources Theory, Poverty, Economic Hardship, Structural Hardship, Psychological Stress, Differential Involvement Theory, Differential Selection Theory, Racial Disparities

## Abstract

Women’s incarceration rates have increased dramatically over recent years; with Black women’s rates disproportionately and significantly higher than other races. Researchers have attempted to understand this criminal justice involvement disparity, and have suggested two major theoretical pathways Differential Involvement and Differential Selection Theories to explain these racial differences. We use the Differential Involvement Theory as a framework to discuss how the objective experience of economic disadvantage as measured by indicators of structural hardship including educational and employment under-attainment and the experience of psychological stress related to resource loss (because of this disadvantage) may explain women’s engagement in criminal activity. In order to conceptualize psychological stress, we used Hobfoll’s Conservation of Resource’s (COR) Theory and measure. Next, we investigated the link between these factors and the degree (number of times incarcerated, number of months incarcerated in lifetime) of criminal behavior using baseline data collected from a NIH study that drew from a racially diverse sample of former substance abusing, criminally involved urban women. Results indicated potential racial differences in the perception of resource loss, and underscore the complex interaction of the experience of race, poverty, and the unique experience of stress on women’s decision making and criminal justice involvement.

## 1. Introduction

Racial disparities in both men’s and women’s criminal justice system involvements are clearly documented; where ethnic minorities are vastly overrepresented in jail and prison populations [1, 2, 3, 4]. Additionally, these racial differences can be found at every step in the criminal justice process; such that compared to Whites, Blacks are more likely to be stopped and held for questioning, arrested, charged, convicted, and given harsher penalties after conviction [4, 6].

Racial disparities in criminal justice involvement are even more striking when one considers women‘s incarceration. Recent reports suggest that women‘s incarceration rates have risen 108% from 1981–2000, compared to an only 78% increase in men’s incarceration rates during the same time period [4, 7,8, 9, 10, 11, 12]. Furthermore, these increased incarcerations disproportionately affect Black women, where 1 out of every 18 Black women can expect to be incarcerated during her lifetime, compared to only 1 out of every 111 White women [4].

This manuscript attempts to explore the racial differences in women‘s American criminal justice involvement. In order to accomplish this task, we will first review the Differential Involvement and Differential Selection pathways to criminality, two respected theories that attempt to explain racial disparities in the criminal justice system. Next, we attempt to apply the Differential Involvement Hypothesis as a theoretical framework to understand how individual‘s structural hardship (economic disadvantage) and psychological stress may explain racial differences in criminal histories. In order to do so, we use Hobfoll’s Conservation of Resources (COR) [13, 14] Theory and measure to tap into individual‘s perception of psychological stress related to economic and social disadvantage. Finally, we present baseline data from a NIH Center for Minority Health and Minority Health Disparities (NCMHD) funded investigation of women with recent criminal justice system involvement, then explore whether race, structural hardship, and perceptions of stress related to this hardship can predict women‘s criminal histories.

The Differential Involvement and Differential Selection Hypotheses [15, 16, 17, 18] both attempt to explain racial differences in criminal involvement. These pathways have important societal implications because they attribute criminal justice involvement to either person-related, or policy and policing-related causes. For example, the Differential Involvement Hypothesis posits that racial differences in the criminal justice system arise because Blacks engage in more criminal activity than non-Blacks [18]. Alternatively, the Differential Selection Hypothesis suggests that Blacks and Whites are just as likely to engage in criminal behavior, however, due to macro level factors (e.g. increased patrolling in predominantly African American poverty stricken urban, neighborhoods), and racism in the criminal justice system, Blacks are more likely to be arrested and processed.

Very few studies have explored these two hypotheses, using female only populations [18, 19]. However, the few studies that do, illustrate the undeniable fact that the intersection of race, gender, and social constructs must be considered. Consequently, Feminist psychologists suggest that racial disparities arise because of the commonly held assumption that women carry a greater societal responsibility as mothers and nurturers in American society, thus, women‘s criminal involvement has steeper societal costs to both family and community [7]. Consequently, perceived non-adherence to these expected norms and gender roles may influence the likelihood of arrest, and of continued involvement in the criminal justice system, particularly for Black women.

Enos investigated racial differences in the sentencing patterns of women detainees, and found that minority women receive harsher penalties, and surmised that these penalties may be due to paternalistic or chivalry-related beliefs of judges and other court officials [19]. Specifically, he suggested that many White middle-aged men with arresting or judicial making power may have tendered less severe punishments to middle-class White women because they outwardly fit the traditional nurturing gender role stereotype [20, 21]. Conversely, women judged not to fit this stereotype may be treated more harshly in the criminal justice process.

Interestingly, Enos also highlighted the connection between poverty and the Differential Selection Hypothesis, and suggested that Black women are more likely to live in disadvantaged, crime ridden neighborhoods that have greater law enforcement presence [19]. Chauhan, Reppuci, Burnette and Reiner found an interesting link between neighborhood disadvantage and disproportionate minority contact (DMC), when they investigated the factors that linked neighborhood disadvantage and antisocial behavior among previously detained female juvenile offenders [15]. Specifically, Chauahan and colleagues first found that Black females were more likely to get arrested for non-violent crimes than Whites [15]. However, interestingly, these results were attenuated when the effects of neighborhood disadvantage (e.g., percentage of people living below the poverty line, percentage of people on public assistance, percentage of women headed households), a proxy for economic community disadvantage, were considered. Once neighborhood disadvantage was added into the model, results indicated that young women living within the most disadvantaged neighborhoods were the most likely to be rearrested.

The first author of this paper is a social psychologist, and is most interested in understanding the complex social cognitive factors that may be embedded in the experience of gender and race, particularly those that may eventually be modified by social services programming. Thus, even though both of these frameworks appear to have explanatory value, our paper utilizes the Differential Involvement Hypothesis to understand racial and gender disparities in criminal behavior engagement only (using participant‘s list of charges as illustrative proof that they engaged in the behavior, and understanding that this behavior may be under reported). Our project is different from previous research, as it draws from an exclusively female sample that reflects most closely the highest criminal justice-involved group of women in the United States: Urban, predominately Black, and former substance abusing.

We think one possible way to understand minority women’s increased risk for engagement in criminal behavior lies in minority women’s experience of poverty and resource loss. Economic disadvantage, poverty, and substance abuse are clearly linked to criminal justice involvement [2, 18,23, 24, 25, 26], and given that minority women are disproportionately affected by poverty [25], it is no surprise that minority women are also substantially more likely to become involved in the criminal justice system. Furthermore, research has shown that although there is a significant rise in aggressive criminal acts for women [9] the majority of women‘s criminal activity is economically driven and conducted to gain or maintain access to economic/employment, housing, or childcare resources through fraudulent means [4, 9, 22]. However, one cannot assume a strict one-for-one relationship between level of poverty and criminal engagement, where poor individuals inherently commit crimes. Thus, it is reasonable to assume that other psychological or social factors may play a role in criminal behavior decision making including adherence to social norms, expectations, and the experience of psychological stress. This project investigates the combined roles of objective structural hardship and the experience of psychological stress related to resource loss on criminal history.

Hobfoll’s Conservation of Resources Theory (COR) [12, 13] conceptualizes the stress that individuals may feel when they encounter resource loss. Resources are defined as those objects, personal characteristics, conditions, or energies that are valued by the individual or that serve as a means for attainment of these objects, personal characteristics, conditions or energies [12, 13]. COR theory is a motivational stress theory that incorporates both the objective and perceived environment in response to stress, and explains how stress directly and indirectly influences health and health behaviors [12, 13]. COR theory assumes that people strive to obtain, retain, protect, and nurture resources, and what is threatening to them is the potential or actual loss of these valued resources. Thus, individuals experience psychological stress when there is an actual or perceived a) threat of a net loss of resources b) net loss of resources or c) a lack of resource gain following the investment of resources.

COR Theory also asserts that resource losses may be especially powerful for those individuals that have very few resources at the start, and cautions that these individuals may be especially likely to encounter “loss spirals” where the loss of a key resource (e.g. employment, transportation, romantic partner support, child care) may translate into the loss of several other resources. Furthermore, COR theory suggests that it is these individuals, those that have encountered the most resource loss in the form of loss spirals, who may be especially likely to engage in resource generation schemes that may be shortsighted and prone to failure (e.g. resorting to criminal activity). Thus, this exploratory analysis investigates the relationship between COR loss, race, and other indicators of socioeconomic disadvantage and criminal behavior. We expect a predictive relationship between structural hardship, resource loss, and level of criminal justice involvement.

## 2. Materials & Methods

### Participants

Participants were from a large metropolitan area in the United States who enrolled in a National Institutes of Health, Center of Minority of Health and Health Disparities (NCMHD) longitudinal study designed to examine housing trajectories among formerly criminal-justice involved (within the past two years) women who were also in substance abuse recovery. Among a baseline sample of 200 participants, the average age was 39.94 (SD = 8.58) years. Most women were Black (N = 149; 74.5%) and White (N = 45; 22.5%). Only four (2.0%) women identified as Latina, while two women were ‘Other’ (N = 2; 1.0%). The over-representation of African American women in this sample reflects the typical criminal justice system rolls in large urban cities, and allows us a large enough sample size to truly investigate how the experience of race may intertwine with both objective measures of structural hardship and the social cognitive perceptions related to the experience of stress related to this disadvantage on women’s pathways into criminal behavior (type of crime committed, total time incarcerated, number of incarcerations).

More than half of the sample had completed high school, GED, or college (N = 119; 59.5%). On average, women were mothers to 2.8 children (SD = 2.25). Less than half of participants reported being employed immediately prior to their last arrest and incarceration in county jail or prison (N = 68; 34.0%). However, most women (N = 171; 85.9%) reported that they had some source of steady financial support the year prior to their last incarceration. At the time of the baseline interview, one-fifth of the sample were awaiting charges, trial or sentencing (N = 42; 21.0%), and more than half were on probation or parole (N = 115; 57.5%; See Table 1 for sources of participant demographics and other criminal history variables). Participants reported a lifetime average incarceration time of 46.30 months (SD = 69.70), with their most recent incarceration time of an average of 12.28 months (SD = 42.65).

### Measures

#### Participant demographics and other criminal history

In addition to standard demographic information (race, ethnicity, and age), participants answered questions about their employment, criminal, legal, family, and substance use histories. For example, specific questions inquired about the total lifetime incarceration, and time since last incarceration (both coded in months), as well as the number of arrests and names of the charges for various criminal offenses. Participants also answered questions about the sources of their financial support prior to arrest (Reported in Table 1).

For the purpose of this study, race was recoded into Black (N = 149) and White, including Latina and ‘Other’ (N = 51). Educational attainment was also recoded into a dichotomous variable to allow for comparisons between high school graduates (N = 119), and those who had not completed high school (N = 81). Additional questions were drawn from subscales of the Addiction Severity Index (ASI) [26], a widely used, valid, and reliable measure employed for both treatment planning and program evaluation among samples of active and former substance users.

#### Conservation of Resources

The original COR-E [12] scale contains 74 key resources to evaluate individual perceptions of loss across several types of resource domains. For the purpose of this analysis, a short form (45-item) of the original measure was used. Participants were asked to rate how much they perceived a loss or the threat of loss in the past three months using a 5-point Likert scale with 1 = Not at all and 5 = Greatly. The COR-E is presented as a checklist, and includes items such as ‘personal health,’ ‘adequate food,’ ‘stable employment,’ and ‘feeling that I am successful.’ Given the checklist properties of the COR-E, an examination of the reliability of the measure is not warranted [27].

### Procedure

Eligible participants were 1) women living in an urban Midwestern city who had been criminal justice system involved within the past two years, 2) had self-identified as former substance abusers at the time of their arrest, and 3) had reported a commitment to continued substance abuse abstinence. Potential participants were recruited from the county jail and substance abuse treatment programs as they exited jail or treatment. Interested participants were enrolled into a two-year longitudinal project with follow-up interviews performed every six months. Prior to enrollment, participants provided informed consent and detailed contact information for follow-up interviewing. Then, participants completed baseline interviews that assessed several factors related to the participant’s previous criminal justice, sexual risk taking, substance abuse, social support, mental health, employment, family, and romantic relationship histories. The results reported originate from the data collected during these baseline interviews. Participants were compensated for the baseline interview with $40 gift cards to a local grocery store.

## 3. Results & Discussion

Analyses were conducted to assess if participants’ race predicted degree of criminal justice involvement, using structural hardship (educational and employment under attainment) and Resource Loss (COR-E) as predictors. Race was found to be associated with both the number of times arrested, and number of months incarcerated during one’s lifetime. Specifically, Black women reported significantly more arrests than White women (*t (188.27) = 3.51, p< .01*), where Black women had an average of 20 arrests/charges (SD = 39.87), and White women only reported an average of 7 arrests (SD = 9.94) in their lifetimes. Additionally, Black women reported significantly longer periods of lifetime incarceration (*t (167.45) = 3.08, p< .01*) where Black women spent an average of 53 months (SD = 76.40) incarcerated, as compared to 27 months (SD = 27.14) incarcerated for White women. Race was also associated with key indicators of structural hardship such that educational attainment differed by race, *x^2^*(1, N = 200) = 10.18, p < 0.01, Phi = 0.23. More White women (78.4%) had obtained at least a high school diploma than Black women (53.0%). A similar trend was identified with employment, *x^2^*(1, N = 200) = 5.20, p < 0.05, Phi = 0.16, where more White women (35.3%) had been employed the six months prior to their incarceration than Black women (20.5%). Interestingly, Black women reported significantly lower levels of resource loss (*M = 83.13; SD = 35.70*) in the past 3 months than White women (M = 98.25; SD = 43.60, t (68.48) = −2.18, p < .05). Race was also not associated with participants’ likelihood to engage in selling drugs, prostitution, or other illegal activities as a way to provide their own steady source of financial support prior to incarceration. Finally, race was not significantly associated with reliance on public assistance (welfare, disability), given that both Blacks and Whites were just as likely to receive financial support from these avenues.

Regression analyses were conducted to investigate the relationship between race, criminal justice variables, and resource loss. Race significantly predicted the number of times arrested and charged (R^2^ = 0.02, F(1, 199) = 4.86, p < 0.05) such that White women reported 12.46 less arrests/charges than Black women (B = −12.46; SE = 5.65; β = −0.16; CI = −23.61—1.32), t(199) = −2.21, p < 0.05). Race also predicted how many months someone was incarcerated in her life (R^2^ = 0.03, F(1, 199) = 5.28, p < 0.05) as White women reported 25.71 less months incarcerated than Black women (B = −25.71; SE = 11.19; β = −0.16; CI = −47.77—3.64), t(199) = −2.30, p < 0.05. Race also significantly predicted COR Loss scores (R^2^ = 0.03, F(1, 196) = 5.82, p < 0.01) such that White women scored 15.12 points higher than Black women (B = 15.12; SE = 6.27; β = 0.17; CI = 2.76–27.47), t(196) = 2.41, p < 0.05. Furthermore, race remained a significant predictor of the number of times arrested and charged with offenses (R^2^ = 0.03, F(1, 196) = 2.69, p = 0.70), and number of times incarcerated when accounting for COR loss scores (R^2^ = 0.03, F(1, 196) = 3.35, p < 0.05). When controlling for COR loss scores, Black women continued to report 11.81 more arrests and charges (B = −11.81; SE = 5.92; β = −0.14; CI = −23.47—0.14), t(196) = −2.00, p < 0.05, and 25.45 more months incarcerated than White women (B = −25.451; SE = 11.67; β = −0.16; CI = −48.48—2.43), t(196) = −2.18, p < 0.05.

For the most part, our preliminary findings are an exact reflection of what current research asserts about the link between race and criminal involvement. Black women report more structural hardships, lower educational attainment, less stable employment, and greater involvement in the criminal justice system, (as indicated by higher numbers of lifetime arrests, and larger incarceration times). Conversely, we did not find a racial difference between the likelihood of women to report engaging in criminal behaviors to generate income, nor the likelihood of women to receive governmental support (disability, public assistance).

We also did not find any racial differences in the type of criminal arrest charges between Black and White women, indicating that both races were equally likely to be arrested for all 11 offenses. Furthermore, we found that most of our sample reported being arrested or charged for non-violent crimes, with only 22% of all charges reported as violent (weapons, homicide, arson, assault, robbery/larceny). Interestingly, this number is higher than reports from previous decades, which may highlight the current research indicating a rise in women’s likelihood to be charged with more aggressive acts [9].

We did find some very interesting results when we considered women’s perceptions of psychological stress over resource loss. Interestingly, it was White women that reported significantly higher COR-E scores. Black women reported significantly lower COR Loss scores, even though these women reported higher actual structural barriers (educational under achievement, unemployment). Thus, it appears as if Black women do not feel as threatened by structural hardships, or resource loss, as White women might. In addition, COR Loss did not predict either indicator of criminal justice involvement (number of total days jailed in life, number of lifetime arrests and charges). However, race was found to predict these indicators, even in models that fist accounted for COR Loss scores.

The discordance in these two findings led us to consider what intervening variables may have influenced our findings. Given that Black women in our sample did not report heightened levels of resource loss, we can assume one of two scenarios. First, we may assume that the Black women in our sample did not suffer from as great of a loss of resources in the time period as White women. Alternatively, we could also assume that our subjective measure of resource loss may be an inaccurate reflection of structural disadvantage for the Black women in our sample. The COR Loss measure assesses the perception of loss of a given set of resources in the past three months. Thus, this measure assumes that individuals have had access to these valuable resources in the first place. Perhaps, this assumption of “having” resources may be the reason for Black women’s lowered COR Loss scores. As Black participants evaluated each of the resources on the list, the stress they felt about losing something they never had (e.g., car, steady employment, health insurance, someone that could loan money if needed) may not have been palpable. Simply put, one may not experience stress over losing things that one may have never had in the first place. Thus, this measure may have failed to capture the complex experience of consistent resource lack and other poverty related conditions that may typify the Black, urban, female experience. Future research should endeavor to understand the effects of chronic resource lack, and resulting psychological distress, on minority women’s risk for criminal justice involvement.

## 4. Conclusion

Our findings present an interesting and complex picture of the relationship between race, perceptions of resource loss, indicators of structural hardship linked to economic instability, and criminal justice involvement. Similar to previous studies, this investigation underscores the obvious, yet complex and intertwined, link between race and the pathway into criminal justice involvement. We utilized a sample often neglected in criminal justice literature, formerly incarcerated women with substance abuse histories. However, this investigation is not without limitations. Firstly, all of the women in our sample were former substance abusers, and had claimed to be in substance recovery upon entering the research study. Thus, these findings may not generalize to samples of criminal involved women without substance abuse histories, or for women that are currently engaged in substance abuse. However, we feel confident that these results are important given that a substantial proportion of women in the criminal justice system are more likely to resemble our sample population than those few that might not.

Our research study also relied on individual self-reports of criminal justice involvement, thus, there is a potential for women to give biased reports on these two indicators. Future studies may choose to include verifiable records of criminal justice involvement to strengthen the validity of these results. Finally, one potential large limitation may be in the way that we measured psychological distress as related to hardship. The COR-E and theory have been designed to capture recent resource loss, rather than chronic lack. It appears that an important racial distinction may exist between these conceptualizations, and delving into them further could lead to a better understanding of racial disparities in women’s criminal behavior engagement. Finally, criminal behavior, and decision making regarding criminal behavior is incredibly complex, and has been shown to be linked to not only these factors, but also to other social cognitive factors inexorably linked to high crime and high poverty neighborhoods, including social norms, self-efficacy, and interpersonal trauma experience. Future research may embed this investigation in research that evaluates these factors. If the Differential Involvement hypothesis rings true for many urban Black women, it is important to understand how we might intervene to reduce the risk of women’s likelihood of criminal decision making. Whether it be via diversion programs that address the psychological and true resource needs, opportunities for education, or by tackling community social norms and beliefs about the acceptability of criminal behavior for women, and the entire urban community; it is our ultimate responsibility to dismantle the factors that cause this radical disparity.

## Figures and Tables

**Table 1:** Participant demographics and other criminal history

Variable	N	%
Source of financial support		
Employment	75	37.5
Ex-Partner	57	28.5
Current Partner	49	24.5
Family	78	39.0
Unemployment	13	6.5
Retirement/Disability	16	8.0
Welfare/Public Assistance	112	56.0
Drug Sales	61	30.5
Trading Sex (prostitution)	71	35.5
Other illegal activities	48	24.0
History of charges		
Shoplifting/vandalism	93	46.5
Parole/probation violations	102	51.0
Drug charges	144	72.0
Forgery	43	21.5
Weapons offenses	20	10.0
Robbery	29	14.5
Burglary/Larceny	30	15.0
Assault	44	22.0
Arson	3	1.5
Arson	3	1.5
Prostitution	69	34.5

## Abbreviations

COR: Conservation of Resources Theory
COR – E: Conservation of Resources Theory – Evaluation (Measure of COR)
NIH: National Institutes of Health
NCMHD – (NIH): Center for Minority Health and Minority Health Disparities
DMC: Disproportionate Minority Contact
ASI: Addiction Severity Index
